# STEM SerialED: achieving high-resolution data for *ab initio* structure determination of beam-sensitive nanocrystalline materials

**DOI:** 10.1107/S2052252523009661

**Published:** 2024-01-01

**Authors:** Pascal Hogan-Lamarre, Yi Luo, Robert Bücker, R. J. Dwayne Miller, Xiaodong Zou

**Affiliations:** a Department of Physics, University of Toronto, 80 George Street, Toronto, Ontario M5S 3H6, Canada; b Max Planck Institute for the Structure and Dynamics of Matter, Hamburg, Germany; c Department of Materials and Environmental Chemistry, Stockholm University, Stockholm SE-106, Sweden; d Department of Chemistry, University of Toronto, 80 George Street, Toronto, Ontario M5S 3H6, Canada; Istituto Italiano di Tecnologia, Italy

**Keywords:** serial electron diffraction, scanning transmission electron microscopy, structure determination, nanocrystallography, beam-sensitive materials, zeolites

## Abstract

Serial electron diffraction (SerialED), which applies a snapshot data acquisition strategy for each crystal, was introduced to tackle the problem of radiation damage in the structure determination of beam-sensitive materials by three-dimensional electron diffraction (3DED). The snapshot data acquisition in SerialED can be realized using both transmission and scanning transmission electron microscopes (TEM/STEM). However, the current SerialED workflow based on STEM setups requires special external devices and software, which limits broader adoption. Here, we present a simplified experimental implementation of STEM-based SerialED on Thermo Fisher Scientific STEMs using common proprietary software interfaced through Python scripts to automate data collection. Specifically, we utilize *TEM Imaging and Analysis* (*TIA*) scripting and TEM scripting to access the STEM functionalities of the microscope, and *DigitalMicrograph* scripting to control the camera for snapshot data acquisition. Data analysis adapts the existing workflow using the software *CrystFEL*, which was developed for serial X-ray crystallography. Our workflow for STEM SerialED can be used on any Gatan or Thermo Fisher Scientific camera. We apply this workflow to collect high-resolution STEM SerialED data from two aluminosilicate zeolites, zeolite Y and ZSM-25. We demonstrate, for the first time, *ab initio* structure determination through direct methods using STEM SerialED data. Zeolite Y is relatively stable under the electron beam, and STEM SerialED data extend to 0.60 Å. We show that the structural model obtained using STEM SerialED data merged from 358 crystals is nearly identical to that using continuous rotation electron diffraction data from one crystal. This demonstrates that accurate structures can be obtained from STEM SerialED. Zeolite ZSM-25 is very beam-sensitive and has a complex structure. We show that STEM SerialED greatly improves the data resolution of ZSM-25, compared with serial rotation electron diffraction (SerialRED), from 1.50 to 0.90 Å. This allows, for the first time, the use of standard phasing methods, such as direct methods, for the *ab initio* structure determination of ZSM-25.

## Abstract 2

An implementation of scanning transmission electron microscopy serial electron diffraction (SerialED) through scripting is proposed. SerialED and continuous-rotation electron diffraction refinement results for the structure of zeolite Y are compared, and the structure of beam-sensitive ZSM-25 is solved by direct methods from electron diffraction data for the first time.

## 1. Introduction

While X-ray crystallography methods remain the dominant tool for atomic structure determination, significant advancements in electron crystallography offer new opportunities for research in chemistry, materials science, structural biology and related disciplines. Historically, structure determination by electron diffraction (ED) relied on manual acquisition of a few zonal-axis diffraction patterns (Dorset, 1996 ▸) and the study of thin 2D crystals (Henderson & Unwin, 1975 ▸). However, the diffraction intensities measured from zonal-axis patterns are affected by multiple scattering (dynamical effects) which limit the application for structure determination, especially for inorganic crystals. In the past 15 years, the development of three-dimensional electron diffraction (3DED) has revolutionized the field of electron crystallography by demonstrating the possibility of routine, reliable structure determination from nanocrystals using off-zone ED data (Gemmi *et al.*, 2019 ▸). Several data collection strategies have been introduced by different groups, now referred to under the umbrella term 3DED, including automated diffraction tomography (ADT) (Kolb *et al.*, 2007 ▸), precession-assisted electron diffraction tomography (PEDT) (Mugnaioli *et al.*, 2009 ▸), rotation electron diffraction (RED) (Zhang *et al.*, 2010 ▸; Wan *et al.*, 2013 ▸), microcrystal electron diffraction (MicroED) or continuous-rotation electron diffraction (cRED) (Nederlof *et al.*, 2013 ▸; Nannenga *et al.*, 2014 ▸; Cichocka *et al.*, 2018 ▸), *etc*. ED techniques have reached a certain maturity, allowing them to complement X-ray structural studies. These techniques have proven successful for structure characterization of many polycrystalline materials that are typically out-of-reach for X-ray diffraction methods owing to small crystal sizes, radiation sensitivity or the presence of multiple phases. These include zeolites (Jiang *et al.*, 2011 ▸; Willhammar *et al.*, 2014 ▸; Guo *et al.*, 2015 ▸; Luo *et al.*, 2023 ▸), metal–organic frameworks and covalent organic frameworks (Feyand *et al.*, 2012 ▸; Zhang *et al.*, 2013 ▸; Wang *et al.*, 2018 ▸; Huang *et al.*, 2021 ▸), proteins and polypeptides (Shi *et al.*, 2013 ▸; Sawaya *et al.*, 2016 ▸; Xu *et al.*, 2019 ▸; Clabbers *et al.*, 2021 ▸), pharmaceuticals (Brázda *et al.*, 2019 ▸; Gruene *et al.*, 2018 ▸; Jones *et al.*, 2018 ▸; Clabbers & Xu, 2020 ▸; Lightowler *et al.*, 2022 ▸), and so forth (Gemmi *et al.*, 2019 ▸).

The 3DED experimental schemes are conceptually analogous to single-crystal X-ray diffraction (SCXRD). Typically, a series of diffraction patterns is collected from an isolated crystal while it is rotated around an arbitrary axis. It is sometimes combined with beam precession (Mugnaioli *et al.*, 2009 ▸) or beam tilt (Zhang *et al.*, 2010 ▸; Wan *et al.*, 2013 ▸). A large rotation range is desired to sample sufficient reciprocal space for structure determination. Although such single-crystal-based 3DED data-acquisition techniques remain important, there are drawbacks when the samples are electron-beam sensitive and lose crystallinity during data collection (Hattne *et al.*, 2018 ▸).

Serial X-ray crystallography can overcome beam-damage accumulation issues in SCXRD by operating in a different paradigm (Chapman *et al.*, 2011 ▸), by which a single still diffraction frame is collected from each randomly oriented crystal of a large ensemble before the crystal is damaged. As each crystal is exposed only once, a much stronger beam can be used for the single snapshot before the onset of damage, leading to a much higher signal-to-noise ratio on each frame compared with those collected by SCXRD.

Similar to serial X-ray crystallography, serial electron diffraction (SerialED) is a technique that applies a snapshot ED data collection strategy to tackle the beam damage problem in conventional 3DED. It was first introduced by Smeets *et al.* (2018*b* ▸), who demonstrated that ED frames from as many as 3500 crystals could be automatically collected per hour on a standard TEM. The random orientation of the crystals on a TEM grid provides a way to stochastically reconstruct the 3DED dataset required to solve the crystal structure. The high signal-to-noise ratio obtained from even extremely sensitive materials comes at the cost of more complex data processing, within which the orientation of each individual crystal has to be determined in order to index diffraction spots on each ED frame. Reflections in all indexed ED frames are then merged into a full dataset. The SerialED data have been used for both *ab initio* structure determination (Smeets *et al.*, 2018*b* ▸) and phase analysis (Smeets *et al.*, 2019 ▸). Later, Bücker *et al.* (2020 ▸) developed a different approach to SerialED, leveraging the capabilities of STEM to achieve a simpler and faster data collection strategy. Together with a thorough redesign of the data analysis, improved control on radiation dose was then demonstrated through a dose fractionation scheme. It enabled a much higher data resolution (1.55 Å) from protein nanocrystals than what is typically obtained by conventional 3DED techniques. This highlights the potential of SerialED in the study of radiation-sensitive materials and nanocrystalline powders.

Innovation in 3DED is closely related to improved instrument control and data collection, which is crucial in designing reliable high-throughput experiments. Many software interfaces for automatic TEM data collection have been developed, especially for biological cryo-electron microscopy with programs such as *Leginon* and *SerialEM* (Suloway *et al.*, 2005 ▸; Mastronarde, 2005 ▸), which have also been adapted to collect cRED data (de la Cruz *et al.*, 2019 ▸; Cheng *et al.*, 2021 ▸). This specifically applies to SerialED, where a large number of crystals have to be addressed by the instrument in fast succession; such features are provided for instance by the Python package *Instamatic* for ED data collection (Smeets *et al.*, 2018*a* ▸). The same system was notably used by Wang *et al.* (2019 ▸) to develop SerialRED for a fully automated workflow for cRED data collection, eliminating the need for human intervention during the experiment (Luo *et al.*, 2023 ▸). Recently, cross-platform software solutions for 3DED were provided as plug-ins to *DigitalMicrograph* (*DM*) (Plana-Ruiz *et al.*, 2020 ▸; Roslova *et al.*, 2020 ▸), a widely used program for TEM control and communication with detectors produced by Gatan Inc. There is major interest in improving the accessibility to advanced electron diffraction techniques, which mandates the development of workflows that can run on any electron microscope with simple integration to existing installations and a high degree of usability.

Herein, we present an implementation of STEM-based SerialED that can be integrated into different S/TEM platforms with minimal apparatus, *i.e.* a Thermo Fisher Scientific (TFS) microscope with STEM and scripting capabilities, and a Gatan or TFS camera. There is no need for implementation of the rather specific hardware implementation employed by Bücker *et al.* (2020 ▸), such as custom scanning coils control, electronic trigger of the beam movement and the camera acquisition, hybrid-pixel detector, *etc*. We first outline the experimental framework of SerialED as well as the motivation for a new implementation. In the following section, we present our software-based solution, from data collection to data analysis. Finally, we show the applications of STEM SerialED for *ab initio* structure determination of two nanocrystalline aluminosilicate zeolites with cubic unit cells, which were chosen to streamline the data acquisition process and ensure high data completeness. SerialED was successfully applied for structure solution of crystals with lower symmetry (Smeets *et al.*, 2018*a* ▸; Bücker, 2020 ▸), despite a relatively low data completeness, especially for crystals with preferred orientations. We first apply STEM SerialED on a relatively stable zeolite Y (FAU framework type) in order to compare the data quality and reliability of structure determination using STEM SerialED with conventional 3DED, more specifically cRED (Cichocka *et al.*, 2018 ▸). We then apply STEM SerialED to ZSM-25, a very beam-sensitive zeolite with a very large unit cell (cubic, *a* = 45 Å, MWF framework type) to demonstrate the advantage of STEM SerialED for *ab initio* structure determination of beam-sensitive materials. The structure of ZSM-25 was previously solved from low-resolution RED data (2.5 Å) by the strong-reflections approach (Guo *et al.*, 2015 ▸) and from SerialRED data (1.5 Å) by a zeolite-specific approach using the program *FOCUS* (Wang *et al.*, 2019 ▸). It was not possible then to obtain high-resolution data from ZSM-25 crystals in order to phase the data by conventional phasing methods such as direct methods.

## 2. Serial electron diffraction: concept

Serial electron diffraction can be conducted in both TEM and STEM modes, referred to here as ‘TEM SerialED’ and ‘STEM SerialED’, respectively. The two methods use different workflows, but remain conceptually similar. In TEM SerialED, the nanocrystal sample is mapped in wide-field TEM mode. Crystals are identified in low magnification with parallel beam illumination. ED data collection on each crystal is then made by switching the microscope between imaging mode and diffraction mode. A parallel electron beam with a predefined size (typically 100–300 nm) is applied to target each crystal. In contrast, the STEM SerialED scheme implemented by Bücker *et al.* (2020 ▸) keeps the microscope in STEM mode throughout the experiment. This approach simplifies the process by only requiring a straightforward switch in beam illumination between focused for crystal mapping and parallel for SerialED data collection. In combination with the beam movement control by the STEM coils (Kolb *et al.*, 2007 ▸; Gemmi & Lanza, 2019 ▸), this significantly improves the beam positioning accuracy and data collection speed. As wide-field illumination is never required, a small condenser aperture (≤50 µm) can be used to allow more parallel illumination during the acquisition of ED frames.

**Figure 1:**
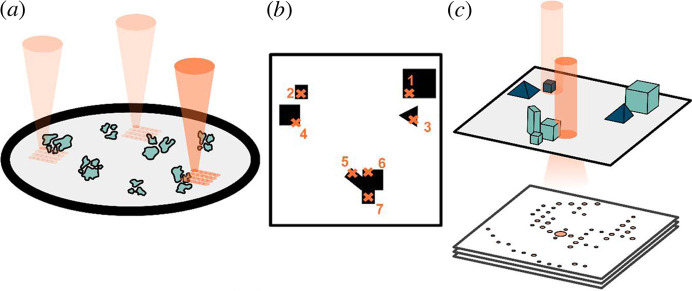
Schematic of a STEM SerialED experiment. (*a*) Search: users manually perform a low-fluence exploration with a field-of-view tens of micrometres across. (*b*) Crystal mapping: users acquire an image of an ROI and identify particles or crystals to target. (*c*) Crystal hopping: via Python commands, selected particles or crystals are sequentially exposed to a collimated beam. An ED frame is recorded for each selected particle/crystal.

SerialED consists of a two-step data acquisition, namely crystal mapping and crystal hopping, described in the following. The entire scheme is presented in Fig. 1 ▸. In crystal mapping, a region of interest (ROI) on a TEM grid, typically tens of square micrometres (*e.g.* 12 × 12 µm), is imaged and used for locating target nanocrystals. Image segmentation is applied to locate target crystals in the acquired image and exclude the grid itself, any unwanted species such as ice crystals, crystalline regions that are too thick, *etc*. Finally, the coordinates of the target crystals are extracted and saved. In crystal hopping, the electron beam moves to target crystals, guided by their coordinates. In preparation, the optical configuration of the microscope is changed by modifying the lens excitation in the projection system (TEM SerialED) and/or the condenser system (TEM SerialED and STEM SerialED). The electron beam is then moved sequentially to the target positions by translating the image coordinates into physical coordinates on the TEM grid. Simultaneously, an ED frame is captured at each position by a detector synchronized to the beam movement. This two-step process is then repeated until enough ED frames from all target crystals in an ROI have been collected. The goniometer is then shifted to expose a new ROI and the same process is applied until all selected ROIs have been investigated. A single SerialED dataset is built from the data of all the selected ROIs.

Although the STEM SerialED workflow reported by Bücker *et al.* (2020 ▸) could achieve high-resolution structure determination in a highly efficient manner, the experimental pipeline relies on the availability of specific equipment. Specifically, a custom arbitrary-pattern STEM generator and a hardware-synchronized detector are required, both of which, while becoming increasingly common with the widespread adoption of 4D-STEM schemes (Ophus, 2019 ▸), are not yet common in standard STEM installations. In order to make STEM SerialED routinely accessible to a much broader user base, we developed a different approach which offers most of the advantages of STEM SerialED, but only requires hardware readily available on a standard STEM. Here, we present the case of a TFS microscope controlled by *TEM Imaging and Analysis* (*TIA*) in combination with Gatan’s *DM*.

## 3. STEM SerialED: implementation

The new STEM SerialED experiments were carried out on an aberration-corrected Themis Z S/TEM from TFS equipped with a Gatan OneView IS camera (16 megapixels, pixel size of 15 × 15 µm) at 300 kV. High-angle annular dark field (HAADF) STEM images were acquired for crystal mapping, using a HAADF detector to collect scattered electron beams at high angles. The open-source Python package *Instamatic* developed in Zou’s group (Smeets *et al.*, 2018*a* ▸), which already gathers the scripting capabilities of various S/TEMs and cameras for versatile experiment design, was used as a toolbox for TEM automatization. Here, we extended *Instamatic* to include control of *TIA*, software commonly used for controlling cameras and STEM acquisition in TFS microscopes. We used *DM* scripting to control the acquisition of ED frames. In its current state, our software solution can be used directly with any TFS S/TEM equipped with either a Gatan detector, a TFS detector or any other detector supported by *Instamatic* (*e.g.* Amsterdam Scientific Instruments’ hybrid pixel detector or TVIPS cameras). This section will cover the experiment control architecture and describe a typical data collection session. Finally, we present the corresponding data analysis by combining our SerialED pre-processing package *Diffractem* (Bücker, 2020 ▸), and *CrystFEL* (White *et al.*, 2012 ▸), which is a widely used data-reduction software developed for serial crystallography.

### 3.1. Experiment control

**Figure 2:**
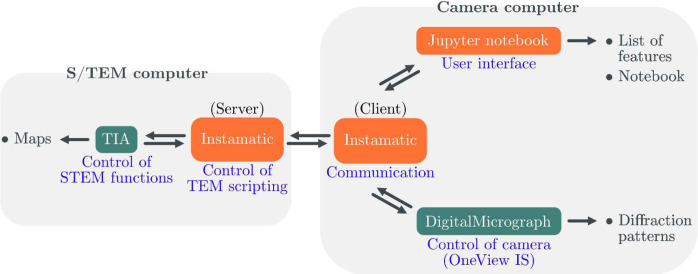
Control pipeline of a typical experiment for STEM SerialED data collection. Python programs *Instamatic* and *Jupyter Notebook* (orange) send directives to proprietary software (green) *TIA* and *DM*. Double arrows represent information flow and single arrows show datafile output for further processing.

We consider a common microscope environment within which *TIA* and *DM* are found on two distinct computers for controlling the S/TEM and the camera, respectively. Although not necessary for the experiment, this configuration isolates computationally intensive tasks from the S/TEM control interface. Fig. 2 ▸ illustrates the control pipeline. The user handles the experiment from the camera computer via *Jupyter Notebook*, which offers interactivity and the possibility to perform some degree of journaling of the experimental session. A server/client connection (TCP/IP protocol) between computers is initiated by a Python script, such that the *Jupyter Notebook* can transmit directives to the S/TEM and receive back status information. With added control over *TIA*, it is possible to acquire, manipulate and transfer STEM images from the *TIA* interface to the notebook. The OneView camera is controlled through a TCP socket connection between *Instamatic* and *DM*, which allows us to execute scripts in *DM* and record ED frames. In recent *DM* versions (GMS 3.4.0 and onward), Python integration enables communication with external programs in a few command lines. However, to maintain compatibility with older GMS versions, we instead opted for the *DM* plug-in *SerialEMCCD* (Schorb *et al.*, 2019 ▸), which serves the same purpose, although initially intended for communication between *DM* and *SerialEM*.

A typical STEM SerialED session is described as follows. The user proceeds with routine STEM alignments for a standard sample, such as an Au/Pd waffle pattern grating (TedPella Inc.). A powder pattern is taken from this standard sample for calibration of the camera length. During data analysis, information from this pattern will be used to decouple the camera length from the unit-cell parameters through a unit-cell refinement step. After these alignments, the condenser system is set to obtain a small parallel beam in nanoprobe mode by only decreasing the excitation of the C3 condenser lens (typically from 74 to 66% on the Themis Z, which corresponds to a defocus of the C3 lens from 0 to −75 µm). This approach facilitates rapid switching of the two optical configurations for crystal mapping and ED frame acquisition. With a 50 µm C2 aperture, we can obtain a beam diameter of 255 nm with a convergence half-angle of less than 0.1 mrad, which is enough to generate sharp diffraction spots. This is very similar to the optical configuration depicted by Bücker *et al.* (2020 ▸). The settings corresponding to the focused beam for imaging (crystal mapping) and parallel beam for diffraction are then stored in the *Jupyter Notebook* to allow rapid switching between the two. The EM grid with the sample of interest is loaded in the STEM and the user explores the grid through *TIA* by collecting STEM images (with a typical size of 10–20 µm), keeping the dwell time of the probe low (≤1 µs) to avoid possible beam damage to the crystals. Once a HAADF-STEM image of an ROI is collected, it is transferred to the notebook in the camera computer for crystal mapping. A Python function segments the map image following user-defined parameters, such as approximate particle size and pixel intensity, as described by Smeets *et al.* (2018*b* ▸). Despite its flexibility, the algorithm might fail to locate target crystals, for example, small crystals and crystals with low contrast. Therefore, there is an option to manually add crystals to the list during crystal mapping using the *TIA* graphical interface with *Instamatic*. Typically, we target about 30 crystals on average for each ROI. This manual selection is imported and added to the algorithmic selection in the steps leading to ED frame acquisition (crystal hopping). Using the *TIA* beam control, the electron probe is moved to an identified position, shortly followed by ED frame acquisition initiated by a *DM* script. These beam movements and ED frame acquisition steps are repeated until all positions in the ROI are addressed, gradually filling up stacks of ED frames that are saved in *DM*. The user resumes their exploration of the TEM grid and repeats all previous steps of crystal mapping and crystal hopping for each ROI encountered. Note that sample height can vary slightly between different regions on the grid. The height change leads to a blurred HAADF-STEM image, which can be compensated by adjusting the defocus of the C3 lens. The *Z*-height of the grid is kept unchanged. In this work, 30 to 50 ROIs were screened per dataset. The number of ROIs needed depends on the purpose of the study, crystal density on each ROI, crystal symmetry, preferred orientation *etc*.

The electron beam and the camera are synchronized in software via a Python script. *Instamatic* is used to communicate between the proprietary software *TIA* and *DM* (Fig. 2 ▸). A new directive is sent to the microscope only when the previous one has been completed. We use pre-specimen beam blanking, which is automatically managed by *DM* and protects both the sample and the camera from beam radiation during the beam movement. Crystals are exposed to the electron beam when the beam is at its target position and the camera is ready to acquire ED frames. Pre-specimen beam blanking guarantees consistency of the fluence from frame to frame in the absence of direct high-speed synchronization between the deflector coils and the camera, as found by Bücker *et al.* (2020 ▸). On the other hand, we find this to be the factor limiting our acquisition speed: with typical exposures of tens of milliseconds, ED frames are acquired at a rate of 0.5–1 frames s^−1^, which is similar to TEM SerialED (Smeets *et al.*, 2018*b* ▸). Without pre-specimen blanking, the acquisition speed becomes primarily limited by the frame rate of the camera itself.

### 3.2. Data analysis

Routine data analysis follows the workflow outlined by Bücker *et al.* (2021 ▸), which uses *Diffractem* and *CrystFEL* alternately. This strategy that combines software takes advantage of the continuous advances in serial crystallography processing, more specifically the development of *CrystFEL*. *Diffractem* offers complementary analysis and provides data in a format appropriate for *CrystFEL*. In brief, data processing comprises the following steps: peak finding, pattern centering, background subtraction, ellipticity correction, indexing, integration and merging. Raw data are first pre-processed by performing peak-finding with the algorithm *peakfinder8* (Barty *et al.*, 2014 ▸). The center of the diffraction patterns, initially estimated as the center-of-mass of the pixels’ intensity, is refined by fitting a Lorentzian profile to the diffuse background and then using the assumption of the symmetry of the peak coordinates around the true center. Background subtraction by radial averaging follows, assuming the background has full rotational symmetry. In parallel, the coordinates of the Bragg peaks are used to characterize the extent of the elliptical distortion (Brázda *et al.*, 2022 ▸), later corrected by modifying the detector geometry in *CrystFEL* accordingly. A virtual powder pattern is generated with the corrected peak coordinates to refine the unit-cell parameters against the camera length deduced from the powder diffraction pattern of the standard Au sample. The pre-processed diffraction patterns with corrected peak coordinates are then indexed either locally or on a remote high-performance computing cluster with *pinkIndexer* (Gevorkov *et al.*, 2020 ▸). Integration and inspection of the results can be performed separately on a local machine. With a satisfactory indexing solution, symmetry-related Bragg peak intensities from all individual ED frames are merged with *partialator* (White, 2014 ▸). The program optionally performs physical modeling by considering beam parameters, scaling, partiality, *etc*. The outcome is a plain-text file enclosing the measured reflection intensities that can be converted for structure solution in *SHELXT* (Sheldrick, 2015*a* ▸).

**Figure 3:**
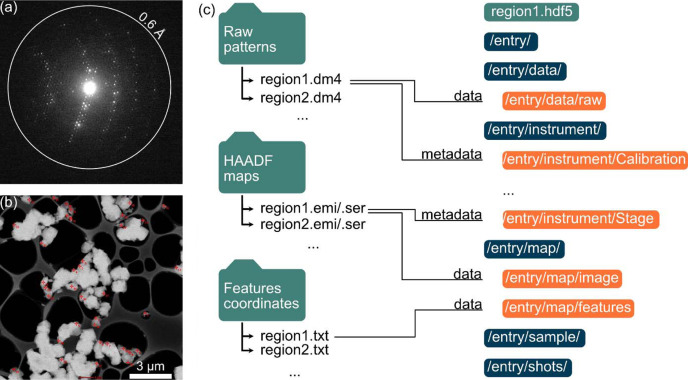
(*a*) Raw ED frame taken off-zone from a zeolite Y crystal, as stored in ‘regionN.dm4’. The white circle indicates a resolution of 0.60 Å. (*b*) Image map of an ROI with manually selected targets marked by red crosses, as stored in ‘regionN.emi’. (*c*) Binary file structure of ‘regionN.hdf5’ to refactor intermediate files in preparation of data preprocessing.

A few changes were made to harmonize this workflow with the current implementation of STEM SerialED. Notably, files produced by *DM*, *TIA* and Python functions are read by a Python script and written back into a single binary file by ROI, following the conventions of *Diffractem* (Bücker *et al.*, 2021 ▸). Based on the Hierarchical Data Format 5 (HDF5), such a file contains all diffraction and mapping data, along with unified metadata of the experiment, all of which are accessible to *Diffractem* for advanced processing. The structure of the binaries is shown in Fig. 3 ▸. In addition to data formatting, the detector panel geometry of the OneView camera needs to be added to *Diffractem*, which was developed primarily on the X-Spectrum Lambda detector based on the Medipix technology (Nederlof *et al.*, 2013 ▸). Multiple detector geometries will be supported by *Diffractem* in time, with minimal modifications required to create new entries.

## 4. Case studies

**Figure 4:**
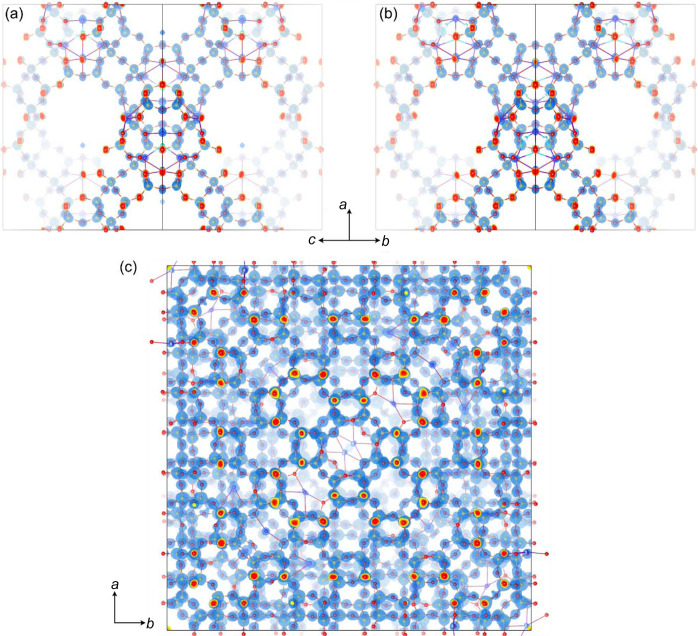
Electrostatic potential maps for zeolite Y and ZSM-25 frameworks obtained from (*a*) STEM SerialED and (*b*) cRED data of zeolite Y with an isosurface at 3σ, and (*c*) STEM SerialED of ZSM-25 with an isosurface at 2σ. The maps were generated using the software *VESTA* (Momma & Izumi, 2011 ▸).

We applied our STEM SerialED to two pure samples of nanocrystalline aluminosilicate zeolites to exemplify structure determination of varying complexity. The first sample is zeolite Y [*Fd* 3 *m*; *a* = 25.0 (2) Å], chosen to investigate the data quality that can be reasonably achieved by STEM SerialED for structure determination of a typical inorganic, microporous material. Our second candidate, as-made ZSM-25 [cubic *I* 43*m*; *a* = 45.0 (1) Å] represents a more significant challenge due to its large unit cell and high beam sensitivity. The two samples are as-made sodium (Na) form materials, and details of the preparation are described elsewhere (Guo *et al.*, 2014 ▸; Guo *et al.*, 2015 ▸). Unit-cell parameters were estimated from the virtual powder patterns created from the STEM SerialED datasets. Crystallographic data and results of structure refinement are presented in Table 1 ▸. The electrostatic potential maps obtained for all structures are shown in Fig. 4 ▸.

### 4.1. Zeolite Y

The framework structure of zeolite Y contains one tetrahedrally coordinated *T* atom (*T* = Si or Al) and four oxygen atoms in the asymmetric unit. STEM SerialED data were collected over ∼4 h from 36 manually selected ROIs that resulted in 1289 raw ED frames. The exposure time was 40 ms per frame and the total electron fluence applied on each crystal during data collection was 0.86 e^−^ Å^−2^ per frame. In total, 358 ED frames were successfully indexed and subsequently merged using partiality modeling, resulting in a dataset with a completeness of 93.0% within a resolution of 0.60 Å. The framework structure was solved *ab initio* by direct methods using *SHELXT* (Sheldrick, 2015*a* ▸), and subsequently refined using *SHELXL* (Sheldrick, 2008 ▸, 2015*b* ▸) with *SHELXLe* (Hübschle *et al.*, 2011 ▸) using atomic scattering factors for electrons (Doyle & Turner, 1968 ▸). All framework atoms (one Si and three O) and two Na^+^ cations in the asymmetric unit could be directly located (Fig. S2 of the supporting information). In the structure refinement, the framework *T* atoms were treated as Si atoms in the refinement because Al and Si have very similar scattering factors. No restraints were applied. The framework atoms and two Na^+^ cations were first refined isotropically to resolve any possible non-framework atoms. One water oxygen (O_w_) was then located in the pore based on the strongest *Q* peak. Finally, all atoms were refined anisotropically without any restraints, and the refinement converged to *R*1 = 0.3010 for 1550 reflections with *I* > 2σ(*I*) and 0.3127 for all 1789 reflections. The chemical composition of the refined structure was determined to be |(Na)_43.1_(H_2_O)_19.7_|[Al_43.1_Si_148.9_O_384_], where the aluminium content was set to balance the positive charge of the Na^+^ cations.

To compare the data quality and refinement results from STEM SerialED data with those from the well-established high-quality cRED data, we collected a cRED dataset from the same sample on a JEOL JEM-2100-LaB_6_ operating at 200 kV with a hybrid pixel Timepix detector. The total angular range for the cRED data collection was 90.20° with 0.233° per frame. The chosen exposure time was 0.5 s per ED frame, and the total electron fluence applied during the entire cRED data collection was 2.3 e^−^ Å^−2^. The cRED data were processed using *XDS* (extended to a resolution of 0.59 Å with an overall completeness of 99.6%). The structure was solved *ab initio* and subsequently refined anisotropically to *R*1 = 0.2200 for 1152 reflections with *I* > 2σ(*I*) and 0.2498 for all 1833 reflections without any restraints.

The bond lengths and angles of the structure obtained from STEM SerialED data are consistent with those determined by cRED, with a mean *T*—O bond length of 1.66 (1) Å for both structures and a mean O—*T*—O angle of 109.47° by STEM SerialED and 109.45° by cRED (Table 1 ▸), which fall between the expected Si—O (1.61 Å) and Al—O (1.76 Å) distances and O—*T*—O angle (109.5°).

Note that weak reflections seem to be more visible in the SerialED data compared with those in the cRED data. However, to meaningfully compare the datasets acquired by different methods, we treated each ED frame in the cRED data individually and processed all frames with *Diffractem* and *CrystFEL* (see Section S3 of the supporting information). The mean *I*/σ for reflections in the higher-resolution shells (0.81–0.60 Å) is higher for STEM SerialED than for cRED (with *I*/σ = 2.42 versus 1.75 at the highest-resolution shell 0.62–0.60 Å). A total of 1550 out of 1789 reflections in the SerialED dataset have *I* > 2σ(*I*), whereas only 1344 out of 1833 reflections in the *CrystFEL*-processed cRED data have *I* > 2σ(*I*) (Table S2 of the supporting information).

### 4.2. ZSM-25

ZSM-25 was chosen to investigate the potential of STEM SerialED for *ab initio* structure determination of challenging electron-beam-sensitive nanocrystals. ZSM-25 was discovered by Doherty *et al.* (1981 ▸). Owing to its large unit cell and small crystal size, the atomic structure of ZSM-25 could only be solved 34 years later using RED data (Guo *et al.*, 2015 ▸). Because ZSM-25 crystals are very beam sensitive, it was only possible to obtain low-resolution (2.5 Å) RED data, and a majority of the reflections were very weak. To obtain an initial structural model from such data, an approach combining intensities of strong reflections of the RED data and the phase information calculated from the structure of the structure-related zeolite paulingite (PAU) was applied. The structural model was then refined and confirmed by Rietveld refinement against synchrotron powder X-ray diffraction (PXRD) data. The chemical composition from the refinement was |[N(C_2_H_5_)_4_]_40_Na_285_(H_2_O)_600_|[Si_1115_Al_325_O_2880_] (Guo *et al.*, 2015 ▸). Later, higher resolution data (1.5 Å) could be obtained using serial rotation electron diffraction (SerialRED) (Wang *et al.*, 2019 ▸), and the structure of ZSM-25 could be solved by a zeolite-specific dual-space method using the program *FOCUS* (Smeets *et al.*, 2013 ▸). However, the resolution was still too low for a structure solution of ZSM-25 by direct methods. Therefore, we chose ZSM-25 to investigate the potential of STEM SerialED for *ab initio* structure determination of very electron-beam-sensitive nanocrystals.

In the present work, STEM SerialED data were collected over a 5 h experimental session from 47 ROIs that resulted in 1364 ED frames on a single EM grid using manual crystal picking. The exposure time was 40 ms per frame and the total electron fluence applied during data collection was 0.66 e^−^ Å^−2^ per frame. After processing, 393 out of the total 1364 diffraction frames were successfully indexed, and the intensity data extracted from the indexed frames were merged by *CrystFEL* into an *hkl* file. The point group *m* 3 *m* was used during data merging to increase data redundancy and ensure accurate modeling of intensities. The data completeness was 99.8% and the resolution cut-off was set at 0.90 Å, in accordance with the statistical criteria that *CC*_1/2_ ≥ 0.15 in the last resolution shell (Karplus & Diederichs, 2012 ▸). The framework structure of ZSM-25 was solved *ab initio* by direct methods using *SHELXT*. It contains 30 *T* atoms (*T* = Si or Al) and 70 oxygen atoms in the asymmetric unit, and all of them could be found. Isotropic refinement was performed using *SHELXL* with the atomic scattering factors for electrons. Bond length and angle restraints were applied for *T* and oxygen atoms. Like for zeolite Y, the framework *T* atoms were treated as Si atoms in the refinement. The structure was refined in the space group *I* 43*m* (217), which converged to *R*1 = 0.2684 for 2888 reflections with *I* > 2σ(*I*) and 0.3351 for all 5740 reflections. Five unique Na^+^ cations could be located in the pores. The chemical composition of the refined structure is |(Na^+^)_156_|[Al_156_Si_1284_O_2880_]. The organic structure-directing agents and water molecules were not included in the structure refinement, and only a fraction of Na^+^ cations were directly located. Compared with RED and SerialRED data, the resolution was greatly improved (from 1.50 to 0.90 Å), and more weak reflections could be observed for the STEM SerialED data, with a mean *I*/σ(*I*) of 2.90 for all reflections and 1.2 even at the highest-resolution shell (1.00–0.90 Å).

## 5. Discussions

The algorithms for intensity integration are very different for cRED and STEM SerialED, with integration of intensities over several ED frames for cRED data and modeling of partial reflections on each frame for SerialED data, respectively. It is therefore important to compare the data quality and the final refined structures obtained from STEM SerialED and cRED data. In the case of zeolite Y, the resolutions of the STEM SerialED and cRED data are similar, 0.60 and 0.59 Å, respectively. This is because zeolite Y is relatively stable under the electron beam, so that the resolution mainly depends on the crystallinity, not the data collection technique. cRED slightly outperforms STEM SerialED based on statistical figures of merit, such as *R* values and completeness. This may also be, in part, due to the fact that the hybrid-pixel detector Timepix provides superior signal-to-noise ratio when used for cRED data compared with the CMOS detector OneView used for STEM SerialED. The accuracy of the structure obtained from SerialED data was evaluated using the program *COMPSTRU* (De La Flor *et al.*, 2016 ▸), which calculates the arithmetic means of the atomic displacements (*d*_ar_) in the asymmetric unit between the STEM SerialED and cRED structures. The *d*_ar_ value is low (0.017 Å with a maximum deviation of 0.039 Å) for the framework *T* and oxygen atoms and significantly higher for the two Na^+^ cations (0.117 and 0.273 Å, respectively). Our results show that the framework structure of zeolite Y refined from STEM SerialED data is very similar to that from cRED data. This indicates that STEM SerialED data are sufficiently reliable for *ab initio* structure determination to obtain accurate atomic structures of inorganic framework materials.

SerialED has a unique ability to capture weak reflections and therefore improved data resolution for very beam-sensitive materials. Based on our analysis with *CrystFEL*-processed cRED data for zeolite Y (Section S3), the fraction of unique reflections with *I* > 2σ(*I*) increased from 73% in the cRED data to 87% in the SerialED data with improved *I*/σ in high-resolution shells (0.81–0.60). Compared with SerialRED data from the beam-sensitive ZSM-25 crystals [1.5 Å resolution (Wang *et al.*, 2019 ▸)], STEM SerialED showed unique advantages to capture both weak reflections and high-resolution reflections from ZSM-25 crystals to achieve a full dataset up to 0.90 Å resolution, which was crucial for the *ab initio* structure solution and refinement. Data quality, especially intensities of reflections at the highest resolution, might be improved if prediction refinement (White *et al.*, 2016 ▸) is implemented in our workflow. It is still challenging to accurately pinpoint both broad low-resolution peaks and weak high-resolution peaks with the current algorithms. A more versatile peak-finding strategy might further improve the results, because neither *CrystFEL* nor the indexer refers to unidentified peaks during prediction refinement.

Based on the high-quality data of ZSM-25 obtained by STEM SerialED, it was possible to distinguish between the centrosymmetric (*Im* 3 *m*) and non-centrosymmetric (*I* 43*m*) space groups. The space group *Im* 3 *m* was applied in previous publications for structure refinement of ZSM-25 against PXRD data (Guo *et al.*, 2015 ▸) and SerialRED data [1.5 Å resolution (Wang *et al.*, 2019 ▸)]. Using the SerialED data (0.90 Å resolution), all framework atoms (Al/Si and O) could be found by *SHELXT* using the space group *I* 43*m*, whereas no reasonable structure solution could be obtained with the space group *Im* 3 *m*. The final *R*1 value from the structure refinement using *I* 43*m* was 0.3350 for all reflections, much lower than that for *Im* 3 *m* (0.5979, Table S1 of the supporting information). Similarly, for structure refinement against the SerialRED data, the space group *I* 43*m* also gave a much lower *R*1 value (0.2639) than *Im* 3 *m* (0.527) (Wang *et al.*, 2019 ▸).

The unit-cell parameter of ZSM-25 first obtained was 44.15 Å, determined from the STEM SerialED data and calibrated using powder rings collected from an Au waffle pattern grating. Considering the uncertainties around the unit-cell determination by SerialED (variation in camera length, indexing error, *etc*.), we used the mean *T*—O distance (1.644 Å) calculated from the ideal distances of Al—O (1.76 Å) and Si—O (1.61 Å) and the Si/Al ratio in the material (3.4, Guo *et al.*, 2015 ▸) for internal calibration of the unit cell (Jones, 1968 ▸). The average *T*—O bond distance in the structure of ZSM-25 refined against the STEM SerialED data using the unit-cell parameter of 44.15 Å was 1.677 Å, which is 2% larger than the expected *T*—O bond distance (1.644 Å). The unit cell was therefore corrected to be 1.644/1.677 × 44.15 Å = 43.28 Å and used for the final structure refinements.

Both the STEM SerialED and the SerialRED data suggest the space group of ZSM-25 to be *I* 43*m*, whereas Rietveld refinement against PXRD data indicates as-made ZSM-25 crystallizes in the space group *Im* 3 *m*. We assume the change of space group can be attributed to the dehydration of ZSM-25 crystals under vacuum in the microscope. This is confirmed by *in situ* PXRD, which exhibits apparent peak shifts to higher angles on heating to 200°C under vacuum. PXRD shows that the unit-cell parameter of as-made ZSM-25 was 45.0848 Å, which shrank to 43.2692 Å when heated to 200°C under vacuum (Fig. S3). The unit-cell parameter obtained from STEM SerialED and corrected based on the *T*—O distance agrees with that of the dehydrated ZSM-25. The profile of the experimental PXRD pattern for dehydrated ZSM-25 also matches the simulated PXRD pattern for the *I* 43*m* structure. This confirms that the structure obtained by STEM SerialED corresponds to the dehydrated ZSM-25. Similar changes in space group and unit cell were also observed for zeolite Na,H-ECR-18 with the PAU framework type (Greenaway *et al.*, 2015 ▸). Na,H-ECR-18 belongs to the same RHO family and has the same cubic space group as ZSM-25, but with a *ca* 10 Å smaller unit cell (Guo *et al.*, 2015 ▸).

With an exposure time of 40 ms and systematic pre-specimen blanking, a data acquisition speed of one crystal per second could be achieved for STEM SerialED, which is comparable to that for TEM SerialED. Major limiting factors on data acquisition speed are searching of ROIs on the EM grid and manual crystal picking, which is sometimes needed. Human interventions can be further minimized with improved algorithms for ROI map segmentation accompanied by the automatic inspection of larger regions of the grid. The latter has been implemented in TEM SerialED and SerialRED using images recorded at systematic stage positions (Smeets *et al.*, 2018*b* ▸; Wang *et al.*, 2019 ▸), and in MicroED using the stitching of images in SerialEM (de la Cruz *et al.*, 2019 ▸). One problem with the current segmentation algorithms for crystal screening was the agglomeration of zeolite nanocrystals, which resulted in a large fraction of unindexable ED frames collected from multiple overlapping crystals. Manual crystal picking was then used to overcome the limitations of the current segmentation algorithms and increase the hit rate.

Note that if the crystals have preferred orientations, high data completeness may not be achieved when data are collected at the same tilt angle. This can be overcome by collecting SerialED data at different tilt angles. The number of ED frames needed for structure determination depends on the symmetry of the crystals, which is much lower for cubic crystals such as zeolite Y and ZSM-25.

We have, for the first time, shown that STEM SerialED data could be reliably collected using a CMOS detector instead of a hybrid-pixel detector. A sufficiently high signal-to-noise ratio in high-resolution shells could be achieved using an electron fluence of 0.5–1 e^−^ Å^−2^ per frame for STEM SerialED. This is much higher than the electron fluence distributed per frame in our cRED experiment with zeolite Y, as a cumulative 2.3 e^−^ Å^−2^ was applied over 357 frames.

## 6. Conclusions

We present the implementation of STEM SerialED based on the Python package *Instamatic*. This software package is readily implemented on any TFS microscope with a STEM unit and TEM scripting capabilities connected to a Gatan camera. Although not demonstrated here, this work can be extended to other types of cameras compatible with *TIA* or *Instamatic*. We show STEM SerialED can be used for *ab initio* structure determination of beam-sensitive nanocrystalline zeolite materials. Routine analysis of datasets for high-resolution *ab initio* structure solution and refinement is possible with the Python package *Diffractem* (Bücker *et al.*, 2021 ▸), and standard X-ray crystallography software (*e.g.* *CrystFEL*, *SHELXT*, *SHELXL*). We verify the accuracy of STEM SerialED by direct comparison of the structures of zeolite Y retrieved by STEM SerialED and cRED under similar conditions. The mean and maximum deviations of the framework atoms are 0.017 and 0.039 Å, respectively, representing 1.0 and 2.4% of the *T*—O bond length. We demonstrate the advantages of STEM SerialED for studying highly beam-sensitive materials using the zeolite ZSM-25. The high-resolution STEM SerialED data (0.90 Å) allows for *ab initio* structure determination of ZSM-25 for the first time by standard phasing methods such as direct methods. Our current implementation for STEM SerialED provides easy access to the technique for crystal structural studies. More automation is desirable to shorten the data-acquisition process, primarily for crystals with low symmetry or preferred orientation. However, with straightforward improvements, it has the potential to become a go-to tool in the study of nanocrystalline powders, particularly for very beam-sensitive materials.

## 7. Code and data availability

The program *CrystFEL* is available from https://www.desy.de/~twhite/crystfel/ under the terms of the GNU general public license. The Python package *Diffractem* is available at https://www.github.com/robertbuecker/diffractem or https://doi.org/10.5281/zenodo.5888864 under the terms of the MIT license. The python package *Instamatic* is available at https://doi.org/10.5281/zenodo.5175957, and modifications for control of *TIA* can be found at https://github.com/phoganlamarre/instamatic/tree/tia. All raw data used in this work are available online at https://doi.org/10.5281/zenodo.10073890.

## Supplementary Material

Crystal structure: contains datablock(s) FAU_cRED, FAU_SerialED, ZSM-25_I-43m_SerialED_calibratedUnitCell. DOI: 10.1107/S2052252523009661/of5002sup1.cif

CIF for refined structures that only serve as comparison in the supplementary materials (e.g. structure determination with an incorrect space group). DOI: 10.1107/S2052252523009661/of5002sup2.txt

Supporting figures and tables. DOI: 10.1107/S2052252523009661/of5002sup3.pdf

Raw data from the STEM used in this work for structure determination.: https://doi.org/10.5281/zenodo.7844520

CCDC references: 2306126, 2306127, 2306128

## Figures and Tables

**Table 1:** Crystallographic data and refinement statistics Due to the close atomic numbers of Al and Si atoms, it is challenging to distinguish them. Therefore, all *T* (*T* = Al, Si) atoms were treated as Si atoms in the refinement. The positive charge of Na^+^ cations is balanced by the introduction of the Al atom into the framework. For STEM SerialED datasets, reflections from the snapshot ED frames are merged using the known point group symmetry to improve the results of the *partiality* modeling algorithm. The *hkl* file obtained contains only unique reflections.

	Zeolite Y (FAU)		Dehydrated ZSM-25 (MWF)
	STEM SerialED	cRED	STEM SerialED
Crystal data			
Formula	|(Na)_43.1_(H_2_O)_19.7_| [Al_43.1_Si_148.9_O_384_]	|(Na^+^)_43.1_(H_2_O)_16.0_| [Al_43.1_Si_148.9_O_384_]	|(Na^+^)_156_|[Al_156_Si_1284_O_2880_]
Space group	*Fd* 3 *m* (No. 227)	*Fd* 3 *m* (No. 227)	*I* 43*m* (No. 217)
Unit cell *a* = *b* = *c* (Å)	25.0	25.0	43.27†
Temperature (K)	293	293	293
Electron wavelength (Å)	0.0196	0.0251	0.0196
Resolution§ (Å)	14.40–0.60 (0.70–0.60)	14.38–0.59 (0.68–0.59)	31.19–0.90 (1.00–0.90)
Completeness (%)	93.0 (91.4)	99.6 (99.8)	99.8 (99.3)
*CC*_1/2_	0.934 (0.442)	0.987 (0.904)	0.991 (0.153)
Unique data‡	1789	1833	5740
Observed Data [*I* > 2.0σ(*I*)]	1550	1152	2888
〈*I*/σ〉	4.07 (2.78)	4.88 (1.32)	2.90 (1.21)

Refinement			
*N*_reflections_, *N*_parameters_, *N*_restraints_	1789, 47, 0	1833, 47, 0	5740, 862, 1803§
*R*1, *wR*2, *S* [*F* > 4.0σ(*F*)]	0.3010, 0.6077, 2.052	0.2200, 0.4966, 1.621	0.2684, 0.5468, 1.57
*R*1, *wR*2 (all data)	0.3127, 0.6136	0.2498, 0.5144	0.3351, 0.5815
Mean values			
Bond length *T*—O (Å)	1.66	1.66	1.64
Bond angle ∠O—*T*—O (°)	109.47	109.45	109.4
Bond angle ∠*T*—O—*T* (°)	141.7	141.9	139.8
R.M.S. deviations			
Bond length *T*—O (Å)	0.01	0.01	0.02
Bond angle ∠O—*T*—O (°)	2.4	2.3	2.2

† The unit-cell parameter was calibrated using the average *T*—O distance, corresponding to that of dehydrated ZSM-25.

‡ Values in parentheses correspond to the highest-resolution shells.

§ Because the ratio of the number of unique reflections to the number of parameters is low (5740/862 = 6.65) and nearly 50% of reflections are weak [*I* < 2σ(*I*)], restraints needed to be applied for the *T*—O and neighboring O⋯O distances.
