# Supplementary material for: STEM SerialED: achieving high-resolution data for ab initio structure determination of beam-sensitive nanocrystalline materials
Source: IUCrJ. 2024 Jan 1;11(Pt 1):62–72. doi: 10.1107/S2052252523009661 (PMC10833385; doi:10.1107/S2052252523009661)
Supplement: Supplementary file 3 [file m-11-00062-sup3.pdf]

**IUCrJ**

**Volume 11 (2024)**

**Supporting information for article:**

# STEM SerialED: achieving high-resolution data for ab initio structure determination of beam-sensitive nanocrystalline materials

**Pascal Hogan-Lamarre, Yi Luo, Robert Bücker, R. J. Dwayne Miller and Xiaodong Zou**

## S1. Python interface

...

### Alignment with nanocrystals

#### Focused settings

```
interface.updateComponents()
```

```
#Alignment HAADF
window_name = '13.40.24 Scanning Acquire'
display_name = 'Acquire HAADF Scanning Display1'
image_name = 'Acquire HAADF'
image = interface.components['{}/{}/{}'.
→format(window_name,display_name,image_name)]
plt.imshow(image.data.value)
plt.show()
```

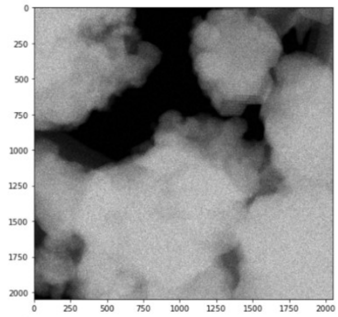

...

```
#Display HAADF
window_name = '21.17.40 Scanning Acquire'#
display_name = 'Acquire HAADF Scanning Display1'
image_name = 'Acquire HAADF'
image = interface.components['{}/{}/{}'.
→format(window_name,display_name,image_name)]
plt.imshow(image.data.value)
plt.show()
```

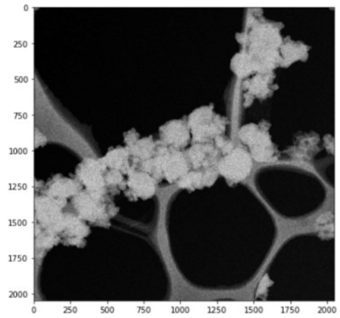

...

### Acquisition

#### Crystal mapping

```
#Particle finder
overview.find_particles(...)
```

```
Found threshold is 15554.1 cts -> binarizing at 35554.1 cts
74 particles found in 50 segments.
```

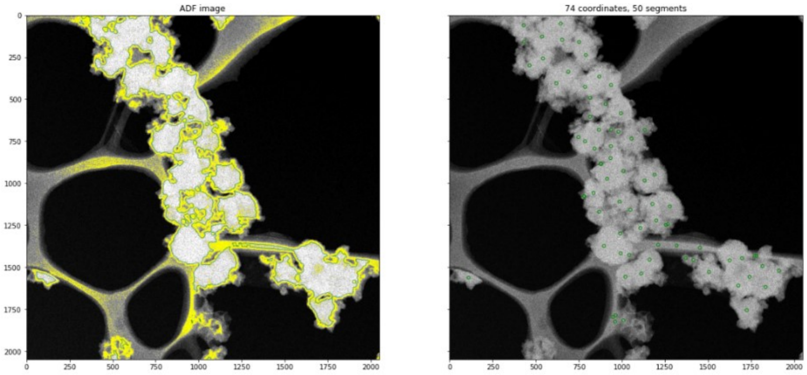


...

**Figure S1** Extracts of the Jupyter notebooks used for STEM SerialED experiments. The format of the document has been simplified and rearranged for the sake of clarity. Text cells show python code. Intermediate steps such as alignment can be documented in the notebook (top). Results from image segmentation and associated scanning beam coordinates (bottom, generated beam positions shown by green circles) are visualized before each acquisition on a ROI.

## S2. Crystallographic results

**Table S1.** Crystallographic details of the structure refinement of ZSM-25 against SerialED data (space groups: *Im*-3*m* and *I*-43*m*) and SerialRED data (space group: *I*-43*m*).[a]

| **Crystal data** | **SerialED** | **SerialED** | **SerialRED**[c] |
|---|---|---|---|
| Formula | $[Si_{1440}O_{2880}]$[b] | $|(Na^+)_{156}|[Al_{156}Si_{1284}O_{2880}]$ | $[Si_{1440}O_{2880}]$ |
| Crystal System | Cubic | Cubic | Cubic |
| **Space group** | $Im-3m$ | $I-43m$ | $I-43m$ |
| Unit cell | | | |
| $a$ (Å) | 43.27 | 43.27 | 43.27 |
| **Data details** | **SerialED** | **SerialED** | **SerialRED**[c] |
| Temperature (K) | 293 | 293 | 293 |
| Electron wavelength (Å) | 0.0196 | 0.0196 | 0.0251 |
| Completeness (%) | 99.8 | 99.8 | 99.7 |
| $CC_{1/2}$ | 98.8 | 99.1 | 99.1 |
| $d_{max}$, $d_{min}$ (Å) | 31.22, 0.90 | 31.19, 0.90 | 18.44, 1.46 |
| Uniq. data | 5407 | 5740 | 2772 |
| Observed Data [$I > 2.0\sigma(I)$] | 2809 | 2888 | 1063 |
| **Refinement** | **SerialED** | **SerialED** | **SerialRED**[c] |
| $N_{reflections}$, $N_{parameters}$, $N_{restraints}$ | 5740, 443, 971 | 5740, 862, 1803 | 2772, 845, 1803 |
| $R1$, $wR2$, $S$ [$F > 4.0\sigma(F)$] | 0.5681, 0.8289, 3.048 | 0.2684, 0.5468, 1.403 | 0.1812, 0.4092, 1.071 |
| *R1, wR2* [all data] | 0.5979, 0.8372 | 0.3351, 0.5815 | 0.2639, 0.4755 |
| Mean values | | | |
| Bond length T-O (Å) | 1.58 | 1.64 | 1.61 |
| ∠O-T-O (°) | 109.4 | 109.4 | 109.4 |
| ∠T-O-T (°) | 145.9 | 139.8 | 143.0 |
| R.M.S. deviations : | | | |
| Bond length T-O (Å) | 0.01 | 0.02 | 0.02 |
| ∠O-T-O (°) | 2.5 | 2.2 | 2.2 |
| ∠T-O-T (°) | 13.8 | 7.8 | 4.8 |

[a] For comparison with the structure refinement of ZSM-25 in space group *Im*-3*m* from SerialRED data, see B. Wang et al. (2019).

[b] Under the space group *Im*-3*m*, we cannot determine the $Na^+$ and $H_2O$ guest species in the structure.

[c] The SerialRED data used for the refinement here were reported by B. Wang et. al (2019).

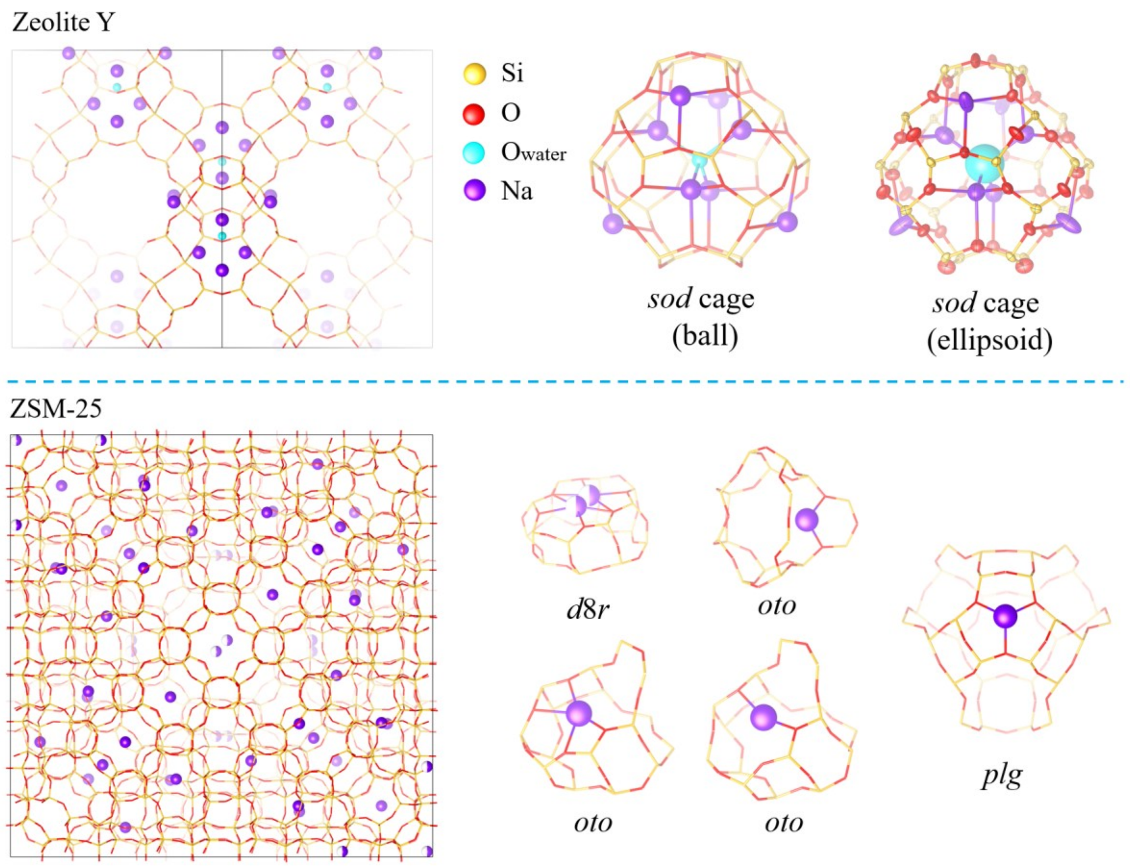


**Figure S2** Structures of Zeolite Y and ZSM-25 determined using the STEM SerialED data. For Zeolite Y, all framework atoms, $Na^+$ cations and water molecules could be located. The $Na^+$ cations and water molecules are found on the surface and in the centre of sod cages. For ZSM-25, with the high-resolution STEM SerialED data, the framework structure in the large unit cell has been directly solved, as well as the positions of $Na^+$ cations.

In-situ PXRD patterns were recorded on a Bruker D8 Advance X-ray diffractometer with Cu Kα as the radiation source at an operating voltage of 40 kV and current of 40 mA (see Figure S3). The patterns are corroborated by the reflections of silicon (marked with *).

Under the vacuum condition (25 °C), the reflections only shifted slightly along the high angle direction compared to the reflections collected in the atmosphere. The unit cell dimensions contracted

from 45.0848 to 44.8243 Å. No obvious changes in the intensities of reflections were observed. These indicate the ZSM-25 was slightly dehydrated and shrank under a vacuum of 4 Pa, but the symmetry of the structure could be maintained. Considering the much higher vacuum in the TEM ($1\times10^{-4}$ Pa), ZSM-25 could experience complete dehydration and thus decrease the symmetry from *Im*-3*m* to *I* -43*m* like the other RHO family zeolites. To confirm this, we performed an additional PXRD experiment on ZSM-25 under a hasher condition (200 °C + vacuum) to dehydrate. After deeper dehydration, the positions and intensities of reflections vary significantly, especially the reflections in the high angle region. The unit cell dimensions further contracted from 44.8243 to 43.2692 Å. The reflection intensities in the high angle region are highly related to the framework structure of ZSM-25. These indicate that dehydration significantly affects the framework structure of ZSM-25 and thus changes the symmetry of the structure. To further confirm that the structural change observed is related to the S/TEM structure, a PXRD pattern for the refined SerialED structure in space group *I*-43*m* was simulated, which matches very well the experimental PXRD pattern of the heated sample (200°C + vacuum).

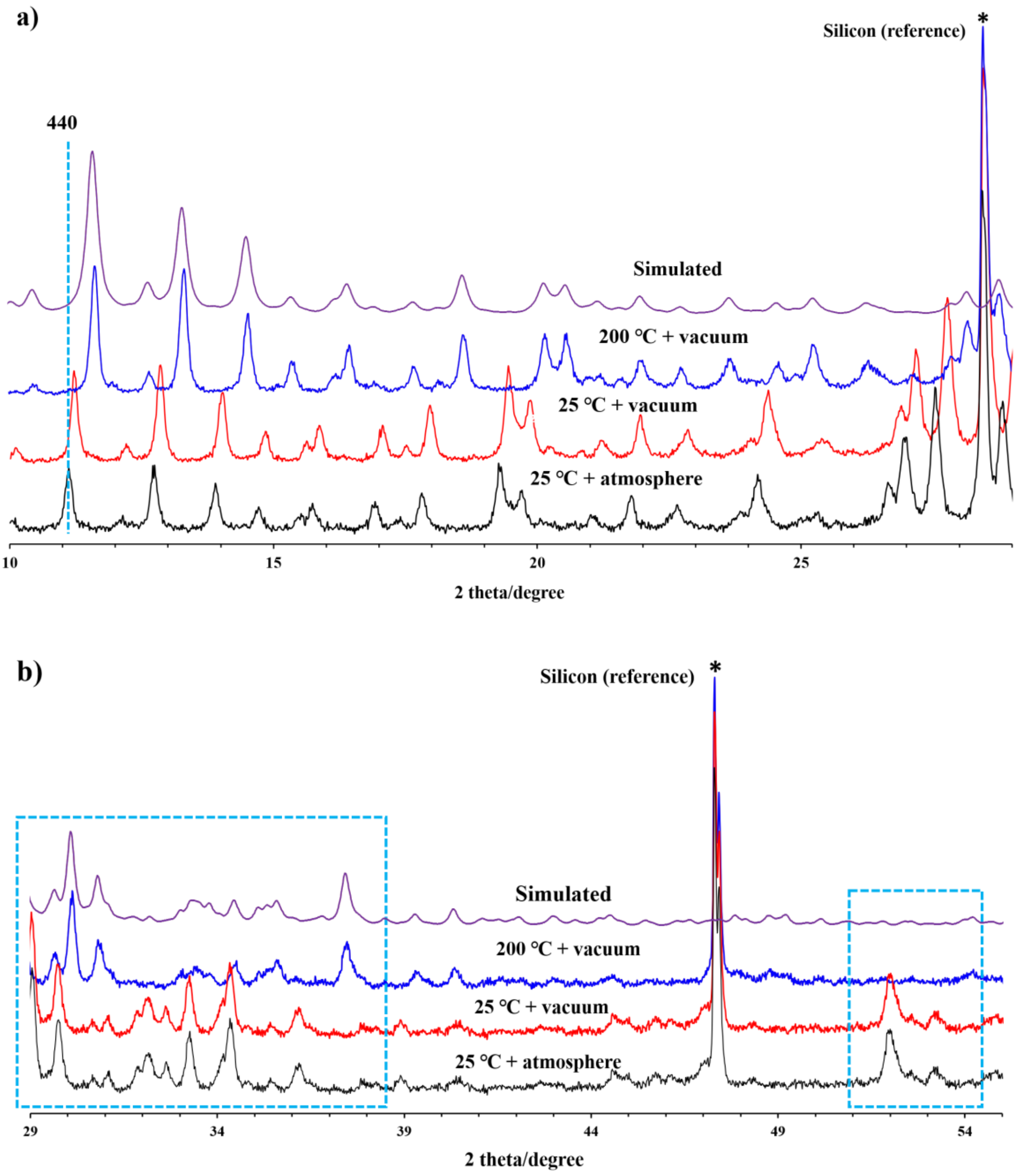


**Figure S3** In-situ powder X-ray diffraction patterns (PXRD, Cukα) of ZSM-25 in low angle region (a) and high angle region (b) recorded under atmosphere, vacuum (4 Pa), and vacuum plus heating (200 °C) conditions. The determined unit cell parameters of ZSM-25 under the three conditions are $a$ = 45.0848, 44.8243 and 43.2692 Å, respectively. The uppermost curve represents the simulated PXRD from our ZSM-25 SerialED structure in the space group *I*-43*m* ($a$ = 43.27 Å).

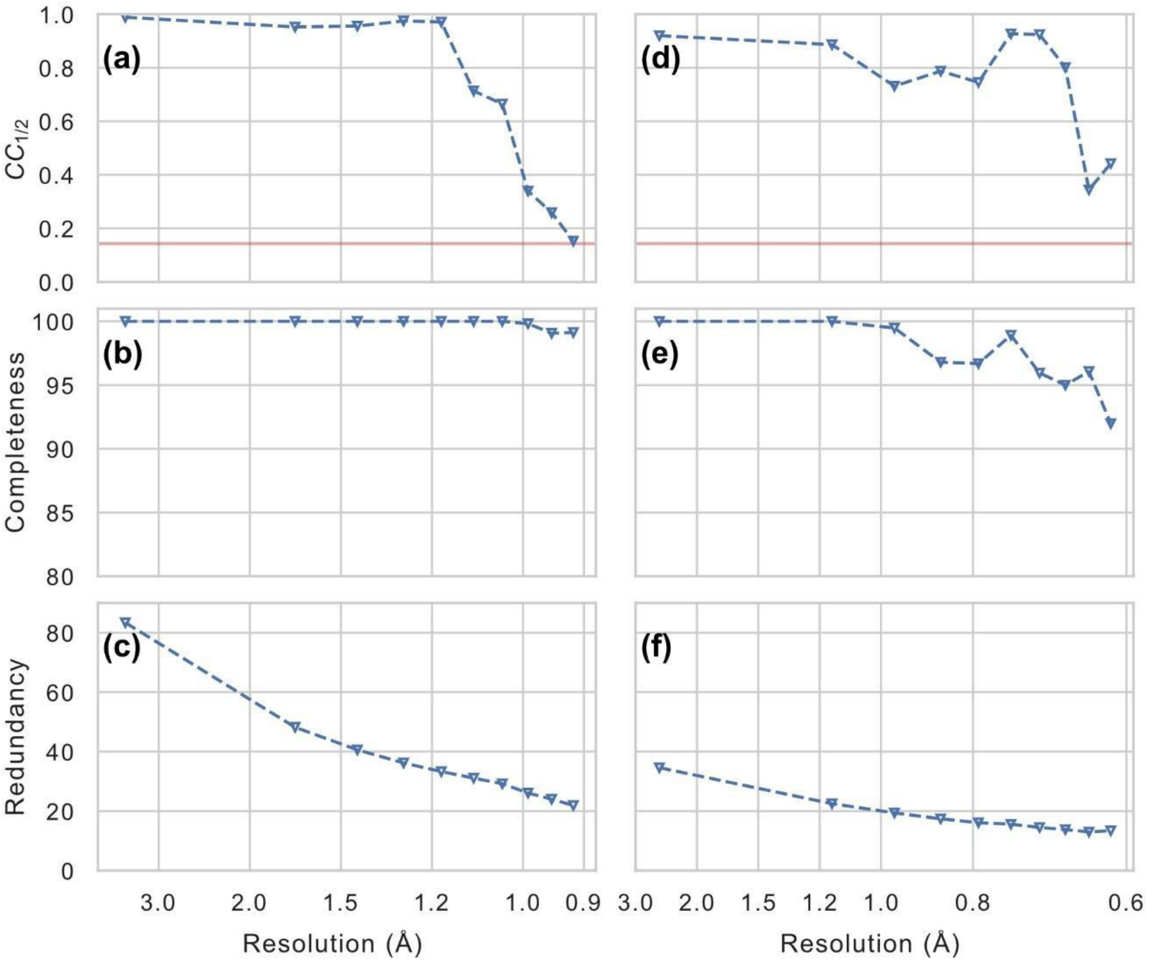


**Figure S4** Variation of selected figures of merit as a function of resolution shell for the final merged datasets of (a)-(c) ZSM-25 and (d)-(f) zeolite Y. The horizontal red line in the $CC_{1/2}$ plots indicates the limit of $CC_{1/2} = 0.15$. Calculations were performed by the CrystFEL programs *check_hkl* and *compare_hkl*.

## S3. Comparison of dataset SNR for STEM SerialED and cRED of zeolite Y

Even though STEM SerialED and cRED experiments were conducted using different S/TEMs and detectors, an attempt was made to compare the signal-to-noise ratio (SNR) obtained in each case. Different software suites may compute the integrated intensities $I$ and associated $\sigma$ through different methods. To make a fair comparison of our cRED and STEM SerialED datasets, we considered the ED frames of the cRED dataset of zeolite Y individually and processed them with Diffractem and CrystFEL. The indexing was conducted with pinkIndexer (Gevorkov et al., 2020). The Timepix detector geometry was added to Diffractem. The 357 individual frames from cRED data were parsed from the tiff file format into an HDF5 file, and treated as independent snapshots, as if they were taken from different crystals. All STEM SerialED processing steps were applied successfully, 351 out of the 357 frames could be indexed. The number of indexed frames is similar to that obtained for the STEM

SerialED zeolite Y dataset (358 indexed frames). With integration and merging in CrystFEL using partiality modeling, we can generate figures of merit computed in the same manner for each dataset with the scripts *check_hkl* and *compare_hkl*. We performed a merging using the point group (*m*-3*m*) to maximize the accuracy of the merging process (e.g., partiality modeling, scaling, etc.). Figure S5 shows the variation of SNR as a function of resolution for the different zeolite Y dataset. STEM SerialED gives higher I/σ over the resolution shell (0.81-0.60 Å). The overall I/σ calculated by CrystFEL is similar in each case: 3.93 for the cRED data and 3.99 for the STEM SerialED data. The I/σ values in the last resolution shell (0.62-0.60 Å) are 1.75 for the cRED data and 2.42 for the STEM SerialED data. The cRED data processed and merged using CrystFEL were then used to refine the structure of Zeolite Y, which converged to $R1 = 0.2627$ for 1344 reflections with $I > 2\sigma(I)$ and 0.2845 for all 1833 reflections. Table S2 compares a few key parameters from structure refinements using the different data. We observe that the number of reflections with $I > 2\ \sigma(I)$ is lower in the CrystFEL-processed cRED data than in the XDS-processed data. This is partly due to background removal in Diffractem. The cRED dataset was acquired using a Timepix hybrid-pixel detector, which has a lower read-out noise and better dynamic range compared to the OneView camera used for STEM SerialED data collection.

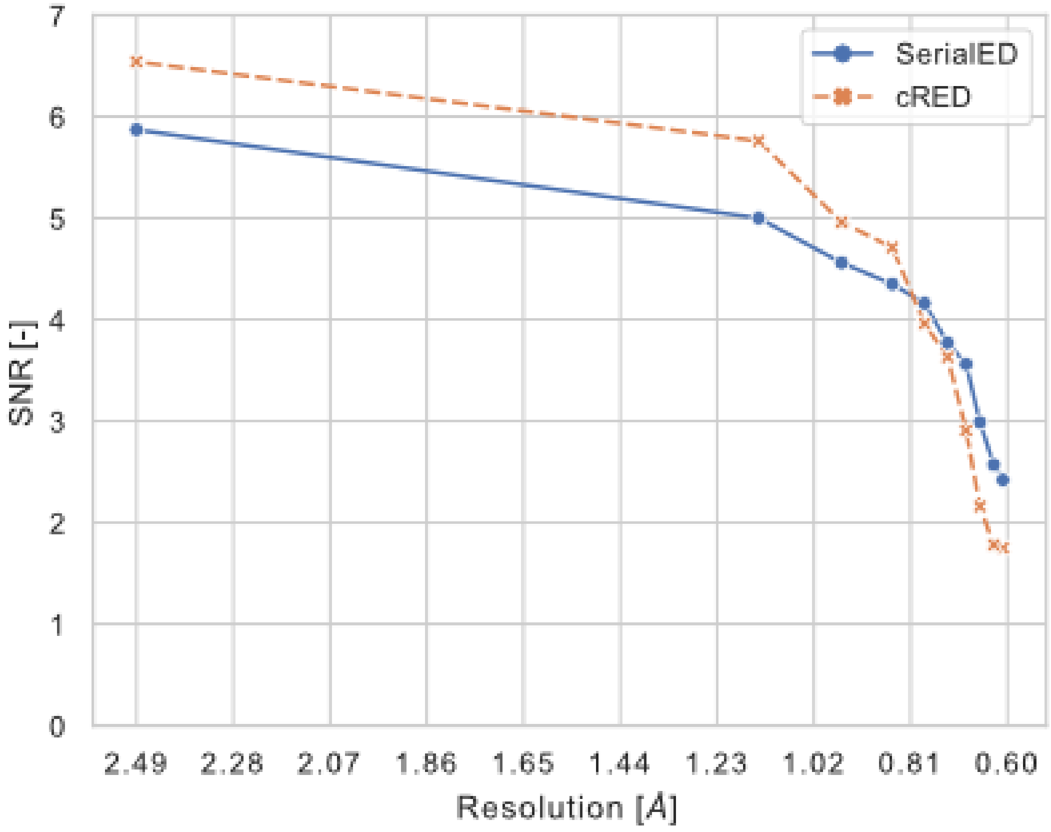


**Figure S5** Variation of SNR as a function of resolution shell for the zeolite Y datasets acquired with different experimental methods. The STEM SerialED and cRED data were collected with different detectors, i.e. the OneView IS camera (CMOS) and the Timepix detector (hybrid-pixel), respectively.

Specifically for this comparison, all data was reprocessed with the STEM SerialED data processing pipeline, including Diffractem and CrystFEL (see section 3.2). Calculations for the SNR were performed by the CrystFEL program *check_hkl* from the hkl files produced by partialator.

**Table S2.** Comparison of crystallographic and refinement values for the structure of zeolite-Y for different data processing and acquisition methods.

| | | | |
|---|---|---|---|
| **Experimental pipeline** | | | |
| Detector | OneView IS | Timepix | Timepix |
| Data acquisition method | SerialED | cRED | cRED |
| Software for frame processing | CrystFEL and Diffractem | XDS | CrystFEL and Diffractem |
| **Data details** | | | |
| Uniq. data | 1789 | 1833 | 1833 |
| Observed Data [$I > 2.0\sigma(I)$] | 1550 | 1152 | 1344 |
| **Refinement** | | | |
| $R1$ [$F > 4.0\sigma(F)$] | 0.3010 | 0.2200 | 0.2627 |
| $R1$ [all data] | 0.3127 | 0.2498 | 0.2845 |
